# Short-term exposure to fine particulate matter and its constituents may affect renal function via oxidative stress: A longitudinal panel study

**DOI:** 10.1016/j.chemosphere.2022.133570

**Published:** 2022-04

**Authors:** Shouxin Peng, Tianjun Lu, Yisi Liu, Zhaoyuan Li, Feifei Liu, Jinhui Sun, Meijin Chen, Huaiji Wang, Hao Xiang

**Affiliations:** a Department of Global Health, School of Public Health, Wuhan University, 115# Donghu Road, Wuhan, 430071, China; b Global Health Institute, Wuhan University, 115# Donghu Road, Wuhan, 430071, China; c Department of Earth Science and Geography, California State University Dominguez Hills, 1000 E. Victoria St, Carson, CA, 90747, USA; d Department of Environmental and Occupational Health Sciences, University of Washington, Seattle, WA, 98105, USA; e Wuhan Center for Disease Control and Prevention, 288# Machang Road, Wuhan, 430024, China

**Keywords:** PM_2.5_, Chemical components, Renal dysfunction, Oxidative stress, Mediation effect

## Abstract

Exposure to fine particulate matter (PM_2.__5_) has been reported to increase the risks of chronic kidney disease. However, limited research has assessed the effect of PM_2.5_ and its constituents on renal function, and the underlying mechanism has not been well characterized. We aimed to evaluate the association of PM_2.5_ and its constituents with kidney indicators and to explore the roles of systematic oxidative stress and inflammation in the association. We conducted a longitudinal panel study among 35 healthy adults before-, intra- and after-the 2019 Wuhan Military World Games. We repeatedly measured 6 renal function parameters and 5 circulating biomarkers of oxidative stress and inflammation at 6 rounds of follow-ups. We monitored hourly personal PM_2.5_ concentrations with 3 consecutive days and measured 10 metals (metalloids) and 16 polycyclic aromatic hydrocarbons (PAHs) components. The linear mixed-effect models were applied to examine the association between PM_2.5_ and renal function parameters, and the mediation analysis was performed to explore potential bio-pathways. PM_2.5_ concentrations across Wuhan showed a slight decrease during the Military Games. We observed significant associations between elevated blood urea nitrogen (BUN) levels and PM_2.5_ and its several metals and PAHs components. For an interquartile range (IQR) increase of PM_2.5_, BUN increased 0.42 mmol/L (95% CI: 0.14 to 0.69). On average, an IQR higher of lead (Pb), cadmium (Cd), arsenic (As), selenium (Se), thallium (Tl) and Indeno (1,2,3-cd) pyrene (IPY) were associated with 0.90, 0.65, 0.29, 0.27, 0.26 and 0.90 mmol/L increment of BUN, respectively. Moreover, superoxide dismutase was positively associated with PM_2.5_ and mediated 18.24% association. Our research indicated that exposure to PM_2.5_ might affect renal function by activating oxidative stress pathways, in which the constituents of Pb, Cd, As, Se, Tl and IPY might contribute to the associations.

## Highlights

• Short-term exposure to PM_2.5_ may affect renal function among healthy adults.
• Ad, Pb, As, Se, Tl and IPY in PM_2.5_ might contribute to the association.
• Oxidative stress might be a bio-pathway between PM_2.5_ and renal function.

## Graphical abstract

**Figure:**
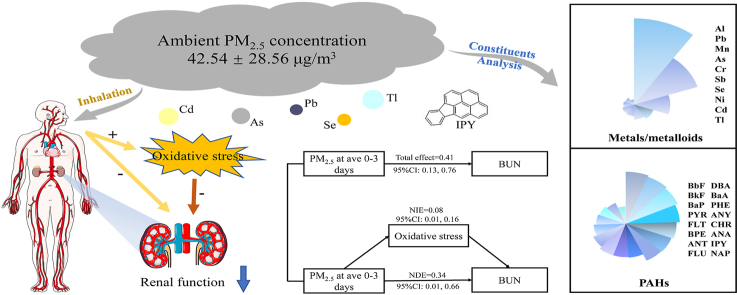

## 1 Introduction

Renal dysfunction has gradually become a global public concern because of the increasing burden of chronic kidney disease (CKD) (Eknoyan et al., 2004; Couser et al., 2011). A study among 12 countries reported that the prevalence of CKD was 14.3% (Ene-Iordache et al., 2016), and it was estimated that 119.5 million CKD patients diagnosed in China (Zhang et al., 2012). Thus, it is urgent to identify the risk factors for reducing disease burden. Recent studies indicated that environment pollution, especially air pollution, may serve as an important risk factor of CKD (Xu et al., 2018; Al-Aly and Bowe, 2020; Stenvinkel et al., 2020). A modeling study estimated that 6.59 million disability-adjusted life years of CKD worldwide in 2017 were attributable to PM_2.5_ pollution (Bowe et al., 2020). It is of great public health significance to explore the adverse effects of PM_2.5_ on kidney and elucidate potential pathogenic mechanisms.

As a metabolic organ that maintains the fluid and acid-base balance, the filtration and concentration function of kidney make it vulnerable to environmental pollutants (Xu et al., 2018). Several pivotal blood renal function parameters were reported to be significantly associated with PM_2.5_ (Rahmani Sani et al., 2020; Wu et al., 2020; Zhao et al., 2020; Kuźma et al., 2021; Li et al., 2021). For example, a cross-sectional study among 2.5 million young adults reported that each 10 μg/m^3^ increment of PM_2.5_ in 3-years average exposure was associated with 0.85% decrease of eGFR (Li et al., 2021). Another research on rodent models found that sub-chronic exposure to PM_2.5_ could lead to elevated blood urea nitrogen (BUN) levels (Tavera Busso et al., 2018). However, most previous research focused on pregnant women (Zhao et al., 2020), children (Liu et al., 2020) or the elderly (Mehta et al., 2016; Fang et al., 2020), while the associations among healthy adults was not fully characterized. Additionally, PM_2.5_ is a heterogeneous mixture with nephrotoxic constituents, which may contribute to the major association between PM_2.5_ and renal dysfunction. Some studies reported that metals and polycyclic aromatic hydrocarbons (PAHs) in PM_2.5_ were related to oxidative damages and heart rate variability (Wei et al., 2009; Wu et al., 2011). However, limited evidence is available for the association between PM_2.5_-bound components and renal function.

The underlying bio-pathways for the association between PM_2.5_ and renal function remain uncertain. Previous reviews reported that exposure to PM_2.5_ may increase oxidative stress (Li et al., 2020) and inflammation (Tang et al., 2020), and these responses were found to be related with renal dysfunction in some populations (Yilmaz et al., 2006; Upadhyay et al., 2011; Correia-Costa et al., 2016). Since kidney is a highly vascularized organ and is susceptible to vascular dysfunction (Lue et al., 2013). Increased states of vasoconstriction and blood coagulation could decrease renal blood flow, further weakening the filtration function of the kidney. An experimental study in the rodent model reported that chronic exposure to PM_2.5_ could trigger inflammation and oxidative stress pathways which contributed to the PM_2.5_ induced kidney injury (Chenxu et al., 2018). Another research in rat model reported that PM_2.5_ may induce early kidney damage by activating systematic inflammation and oxidative stress response (Aztatzi-Aguilar et al., 2016). However, evidence about the underlying mechanisms remain scarce.

Therefore, we designed the current research with personal PM_2.5_ exposure and components measurements to explore their acute adverse effects on renal function parameters among healthy adults. In addition, we explored the possible mediation effect of circulating biomarkers on the aforementioned associations. The results of our research will contribute to the evidence of the association between PM_2.5_ and renal function and serve the potential bio-pathway.

## 2 Material and methods

### 2.1 Study design and participants

The 7th Military World Games were held in Wuhan, China, from October 18 to October 27, 2019. A series of policy measures were implemented to restrict the road traffic and control air pollution during the match. In our previous research (Peng et al., 2022), we recruited 70 college students for 8 rounds repeated measurements of blood samples collection, in-person investigation and physical examination to explore the adverse effects of PM_2.5_. Baseline demographic information, including sex, date of birth, weight, height, education was collected after signing the informed consent. We randomly selected a subset from the previous research for the current research. Briefly, 35 healthy adults were included with 6 rounds follow-up visits including twice in each of the three phases before (from Sep. 16th to Sep. 27th), during (from Oct. 17th to Oct. 28th) and after the Military games (from Dec. 5th to Dec. 16th). We measured individual-level hourly PM_2.5_ concentrations, and collected the venous blood at the 4th day. And participants were required to reported health status (Healthy or Sick), medication use (Yes or No), caffeine and alcohol consumption (Yes or No), exercise (Yes or No) and dietary intake frequency at each follow-up visit. The research design was approved by Wuhan University Medical Ethics Committee.

### 2.2 Exposure measurement

We conducted the personal hourly PM_2.5_ measurements for 3-consecutive day before each physical examination. We performed the HUAWEI individual PM_2.5_ monitor which was designed with low weight for portability based on Beta ray attenuation methods. And the DUSTTRAK™ DRX 8534 (TSI, USA) was used to calibrate personal exposure devices (Peng et al., 2022). Ambient PM_2.5_ samples were collected by a medium-volume sampler (TH-150C, Wuhan Tianhong Environment Protection Industry Co. Ltd., Wuhan, China) with Whatman quartz fiber filters (Whatman International Ltd., Maidstone, UK) and kept individually in a polystyrene box (SF-90BOX, Beijing Safelab Ltd, Beijing, China). The constituents of ambient PM_2.5_, including trace metals (metalloids) and the polycyclic aromatic hydrocarbons (PAHs) measured by Inductively Coupled Plasma-Mass Spectrum with iCAP-Q (Thermo Fisher Scientific, Waltham, MA, USA) and Gas Chromatography-Mass Spectrometry with Trace 1300-ISQ 7000 (Thermo Fisher Scientific, Waltham, MA, USA), respectively. All experimental manipulations were done at the laboratory of Wuhan Center for Disease Control and Prevention and details can be found in the previous research (Mao et al., 2020). Generally, 10 metals and 16 PAHs were measured from PM_2.5_, such as Aluminum (Al), Arsenic (As), Cadmium (Cd), Lead (Pb), Selenium (Se), Thallium (Tl), Indeno (1,2,3-cd) pyrene (IPY), etc. Additionally, we collected the city-level PM_2.5_ concentrations from 2018 to 2020 from Wuhan Municipal Ecological Environment Bureau (http://hbj.wuhan.gov.cn/) to evaluate the effect of restrictive measures during the Military World Games. The hourly concentrations of ozone (O_3_), nitrogen dioxide (NO_2_), sulfate dioxide (SO_2_) and carbon monoxide (CO) was also collected from the nearest air monitoring station (Wuhan Donghu Liyuan).

### 2.3 Blood collection and analysis

Fasting venous blood samples (20 mL in total, including 10 mL EDTA anticoagulated blood and 10 mL non-anticoagulated blood) were collected before 8:00 a.m. at each physical examination. And the centrifuged plasma and serum samples were stored at −80 °C before biomarkers measurement. Renal function indicators including BUN, sCr, and urea acid (UA) were detected by a full-automatic biochemical analyzer (Hitachi 7600, Hitachi Co., Tokyo, Japan). The blood urea nitrogen-to-creatinine ratio (BUN/sCr) was calculated as a commonly clinical renal function indicator. The eGFR was calculated according to the Chronic Kidney Disease Epidemiology Collaboration Equation (Levey et al., 2009), and the values of endogenous creatinine clearance rate (Ccr) were estimated following the Cockcroft-Gault Equation (Cockcroft and Gault, 1976, Winter et al., 2012). The fasting blood glucose concentrations were measured by an automatic biochemical analyzer (Cobas c701, Roche, Japan). The following 5 circulating biomarkers were measured as potential bio-mediators: (i) inflammation: Hypersensitive C-reactive protein (hsCRP), interleukin-6 (IL-6); (ii) coagulation: fibrinogen (FIB); (iii) oxidative stress: superoxide dismutase (SOD); (iv) vasoconstriction: Angiotensin-converting enzyme (ACE). The serum hsCRP and ACE were analyzed through the Beckman Coulter AU5800 (Beckman Coulter Inc., Brea CA, USA). SOD was analyzed using an automatic biochemical analyzer (Hitachi 7180, Hitachi, Tokyo, Japan) with Superoxide Dismutase Kit. The serum IL-6 was assayed by electrochemiluminescence on Roche cobas 8000 (Roche Diagnostics, Mannheim, Germany). The plasma FIB was analyzed by the Clauss method on SF-8200 coagulation analyzer (Beijing Succeeder Technology Inc., Beijing, China). All the biomarkers were analyzed in the laboratory of Wuhan Pulmonary Hospital (Wuhan, China).

### 2.4 Statistic analysis

Demographics, biological indicators and PM_2.5_ concentrations were described as mean ± standard deviation (SD) or frequency (%). The linear mixed-effect (LME) model with each participant as random intercept was performed to assess the relationship between PM_2.5_ exposure and renal function. We calculated the 1–3 days PM_2.5_ moving average (ave 0–1, ave 0–2 and ave 0–3) to identify the cumulative impact of exposure in the single-pollutant LME model. A group of priori covariates were adjusted in the LME model, including age, sex, body mass index, exercise, caffeine consumption, alcohol consumption, fasting blood glucose. Additionally, the temperature and relative humidity were adjusted in the form of natural splines, while the Akaike information criterion was applied to determine the degrees of freedom. Besides, as previous review reported that high-protein diets serve as a risk factor for renal dysfunction (Ko et al., 2020), we adjusted the high protein proportion food intake (i.e., the consumption frequency of meat, poultry, fish, and milk) in the association between PM_2.5_ and renal function. We also investigated the short-term association between 3-days moving average of PM_2.5_ components and renal function indicators in the single-constituent LME model. The effects were estimated as the changes of indicators and 95% confidence intervals (CIs) with an interquartile range (IQR) increment in PM_2.5_ or its constituents’ concentrations.

We hypothesized that associations of PM_2.5_ with renal function indicators might be mediated through inflammation, oxidative stress, or vasoconstriction. Therefore, we selected SOD, hsCRP, IL-6, FIB and ACE as potential mediators which were suggested to be associated with PM_2.5_ in the previous research. In this study, potential mediators were defined by the following criteria: (i) significantly associated with PM_2.5_ and (ii) significantly associated with renal function indicators (Valeri and Vanderweele, 2013). Two LME models were built for the mediation analysis (Bind et al., 2016), one fitting for the PM_2.5_-mediator association and the other one fitting for the mediator-renal function association (Equations [1], [2])).

[1] *M*_*ij*_ = *β*_*0*_ + *u*_*i*_ + *β*_*PM2.5*_*PM*_*2.5ij*_ + *β*_*1*_*X*_*1ij*_ + … + *β*_*p*_*X*_*pij*_ + *ε*_*ij*_,

[2] *Y*_*ij*_ = *γ*_*0*_ + *g*_*i*_ + *γ*_*PM2.5*_*PM*_*2.5ij*_ + *γ*_*M*_*M*_*ij*_ + *γ*_*1*_*X*_*1ij*_ + … + *γ*_*p*_*X*_*pij*_ + *η*_*ij*_,

In both of two equations, *ꞵ*_*0*_ and *γ*_*0*_ correspond to the intercept for the population mean; *u*_*i*_ and *g*_*i*_ correspond to the subject-specific random intercept. *M*_*ij*_ correspond to the potential circulating biomarkers and *Y*_*ij*_ correspond to the renal function indicators measured for an individual *i* (*i* = 1, …, 35) at visit *j* (*j* = 1, …, 6). *X*_*1ij*_ to *X*_*pij*_ represent the priori-selected covariates, and *ε*_*ij*_ and *η*_*ij*_ represent the within-subject error term. *γ*_*PM2.5*_ represents the natural direct effect (NDE), and the natural indirect effect (NIE) could be given by *β*_*PM2.5*_ × *γ*_*M*_. The proportion mediated, which means the percentage of NIE over the total effect, was calculated by (NIE/(NIE + NDE)).

To examine the robustness of our findings, we performed several sensitivity analyses. Firstly, outcomes of the previous visit might be potential confounding factors for subsequent visits and lead to bias in the longitudinal studies. Therefore, we built a LME model regression between *Y*_*ij*_ (*BUN*_*ij*_) and *M*_*ij+1*_ (*SOD*_*ij+1*_) to examine the time-varying confounding assumption (Fig. S1). Then, we adjusted the other four gaseous pollutants (i.e., O_3_, SO_2_, NO_2_ and CO) into the two-pollutant models. Thirdly, we fitted a “constituent-PM_2.5_ joint model” and “constituent-residual model” to eliminate the extraneous variation of total PM_2.5_ and the collinearity between constituent and the PM_2.5_ mass concentrations (Liu et al., 2017). All the statistical analyses were conducted in the R software (4.0.5) with packages of “lmerTest”, “splines” and “mediation”, and the two-side *p*-value less than 0.05 was determined as statistical significance.

## 3 Results

### 3.1 Descriptive analysis

A total of 35 volunteers (28 females and 7 males) with an averaged age of 20.43 years and a mean BMI of 21.17 kg/m^2^ were recruited in this panel study. And all the participants were nonsmokers. However, 4 participants failed to complete 1 follow-up visit and 1 participant failed to complete 2 follow-up visits for various reasons and thus 6 observations were deleted. Eventually we examined a total number of 204 venous blood samples. Table 1 showed the 12 blood biomarkers levels, including the 6 renal function indicators (BUN, sCr, UA, eGFR, Ccr, BUN/sCr), the 5-potential bio-mediators (SOD, IL-6, hsCRP, ACE, FIB) and the fasting blood glucose. Fig. S2 showed the monthly averaged PM_2.5_ concentrations in Wuhan from September to December for the years of 2018–2020. We found an upward trend of ambient PM_2.5_ concentrations during the 4 months in 2018 and 2020, while the concentrations showed a slight decline in October (during the 7th Military World Games) in 2019. Table 2 summarized PM_2.5_ mass concentrations along with metals and PAHs constituents during the whole research periods. The average individual PM_2.5_ concentrations were 42.54 μg/m^3^, which exceed the Interim Target-2 standard of the WHO air quality guideline on PM_2.5_. Among the various constituents of the PM_2.5_, the metal/metalloid constituents had the higher proportion than PAHs and varied considerably, in which Al, Pb and Mn had large abundant while Ni, Cd and Tl were less.

**Table 1:** Basic characteristics and the biological indicators of study participants.

		Mean ± SD or N (%)
Demographic characteristics		
	NO.	35
	Age, years	20.43 ± 1.74
	BMI, kg/m^2^	21.17 ± 2.59
	Sex, female	28 (80.00%)
Serum and plasma biomarkers		
	FBG, mmol/L	4.61 ± 0.37
	SOD, U/mL	146.79 ± 9.43
	FIB, g/L	2.44 ± 0.41
	hsCRP, mg/dL	0.99 ± 1.31
	IL-6, pg/mL	1.87 ± 1.75
	ACE, U/L	33.00 ± 10.00
Renal function indicators		
	BUN, mmol/L	3.86 ± 1.21
	sCr, μmol/L	63.80 ± 9.50
	UA, μmol/L	341.00 ± 82.00
	eGFR, mL/(min*1.73m^2^)	123.79 ± 10.12
	Ccr, mL/(min*1.73m^2^)	118.63 ± 20.61
	BUN/sCr	15.52 ± 4.74

Abbreviations: SD, standard deviation; BMI, body mass index; FBG, fasting blood glucose; SOD, superoxide dismutase; FIB, fibrinogen; hsCRP, hypersensitive C-reactive protein; ACE, angiotensin converting enzyme; IL-6, interleukin-6; BUN, blood urea nitrogen; sCr, serum creatinine; UA, blood urea acid; eGFR, estimated glomerular filtration rate; Ccr, endogenous creatinine clearance; BUN/sCr, the ratio of blood urea nitrogen to serum creatinine.

**Table 2:** Descriptive statistics of 3-day average ambient PM_2.5_ and PM_2.5_ chemical components for the study participants over the study period.

		Mean	SD	Percentiles			IQR
				25th	50th	75th	
PM_2.5_ (μg/m^3^)		42.54	28.56	23.45	30.25	56.38	32.94
Metals (ng/m^3^)							
	Sb	2.60	1.24	1.77	2.72	3.58	1.82
	Al	148.77	61.89	114.12	131.36	174.55	60.42
	As	6.09	3.54	4.45	5.24	7.27	2.82
	Cd	1.30	0.66	0.87	1.20	1.88	1.02
	Cr	4.43	2.74	3.25	3.37	4.20	0.95
	Pb	65.98	32.55	45.93	65.66	93.66	47.72
	Mn	19.35	5.49	17.46	18.55	20.91	3.44
	Ni	1.67	0.57	1.39	1.82	2.02	0.63
	Se	2.26	0.95	1.62	2.11	2.38	0.76
	Tl	0.51	0.25	0.35	0.50	0.55	0.21
PAHs (ng/m^3^)							
	NAP	0.15	0.06	0.10	0.13	0.17	0.06
	ANY	0.37	0.09	0.37	0.40	0.42	0.05
	ANA	0.29	0.08	0.29	0.30	0.30	0.01
	FLU	0.56	0.20	0.50	0.58	0.61	0.11
	PHE	0.47	0.12	0.45	0.49	0.52	0.07
	ANT	0.82	0.22	0.76	0.86	0.92	0.16
	FLT	0.93	0.29	0.75	0.93	1.12	0.37
	PYR	1.05	0.31	0.99	1.05	1.21	0.23
	CHR	0.31	0.22	0.18	0.21	0.35	0.17
	BaA	0.54	0.18	0.47	0.53	0.61	0.14
	BbF	1.32	0.34	1.23	1.39	1.49	0.26
	BKF	1.25	0.53	1.02	1.10	1.62	0.60
	BaP	1.17	0.33	1.05	1.26	1.43	0.39
	DBA	0.54	0.13	0.43	0.54	0.63	0.20
	BPE	0.85	0.60	0.48	0.62	1.04	0.55
	IPY	0.25	0.20	0.11	0.17	0.41	0.30

Abbreviations: SD, standard deviation; IQR, interquartile range; Sb, Stibium; Al, Aluminum; As, Arsenic; Cd, Cadmium; Cr, Chromium; Pb, Lead; Mn, Manganese; Ni, Nickel; Se, Selenium; Tl, Thallium; PAHs, Polycyclic aromatic hydrocarbons; NAP, Naphthalene; ANA, Acenaphthene; ANY, Acenaphthylene; FLU, Fluorene; PHE, Phenanthrene; ANT, Anthracene; FLT, Fluoranthene; PYR, Pyrene; CHR, Chrysene; BaA, Benzo (a) pyrene; BbF, Benzo (b) fluoranthene; BkF, Benzo (k) fluoranthene; BaP, Benzo (a) pyrene; BPE, Benzo (g,h,i) perylene; DBA, Dibenzo (a,h) anthracene; IPY, Indeno (1,2,3-cd) pyrene.

### 3.2 Estimated association between PM_2.5_ and renal function

**Fig. 1:**
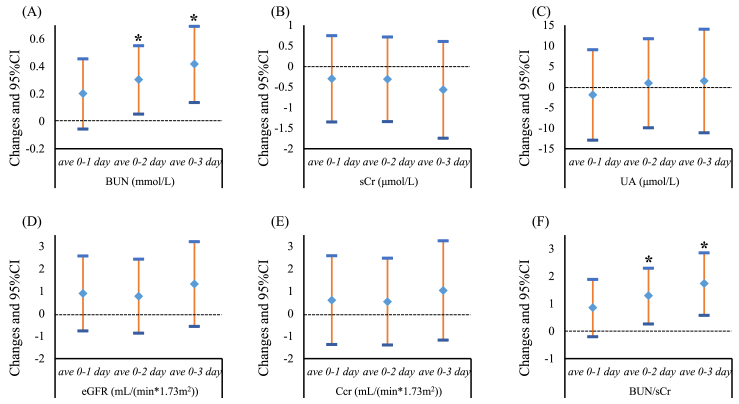
Changes in renal function indicators (mean and 95% confidence intervals) with an interquartile range increment of PM_2.5_ in different exposure windows. (A) BUN, blood urea nitrogen; (B) sCr, serum creatinine; (C) UA, urea acid; (D) eGFR, estimated glomerular filtration rate; (E) Ccr, endogenous creatinine clearance rate; (F) BUN/sCr, blood urea nitrogen-to-serum creatinine. *Estimated were statistically significant (*p*-value < 0.05).

Fig. 1 presented the estimated associations of PM_2.5_ concentrations with renal function indicators (BUN, sCr, UA, eGFR, Ccr and BUN/sCr). Short-term exposure to PM_2.5_ were positively associated with BUN and BUN/sCr. An IQR increase in PM_2.5_ (32.94 μg/m^3^) was associated with 0.30 mmol/L (ave 0–2, 95% CI: 0.05 to 0.55) and 0.42 mmol/L (ave 0–3, 95% CI: 0.14 to 0.69) increment of BUN, respectively. For the BUN/sCr, an IQR increment in PM_2.5_ was associated with 1.29 (ave 0–2, 95% CI: 0.27 to 2.30) and 1.74 (ave 0–3, 95% CI: 0.58 to 2.86) elevated in BUN/sCr, respectively. However, the estimated effect of PM_2.5_ on other renal function indicators were not significant.

### 3.3 Estimated relationship of PM_2.5_ constituents with renal function

**Fig. 2:**
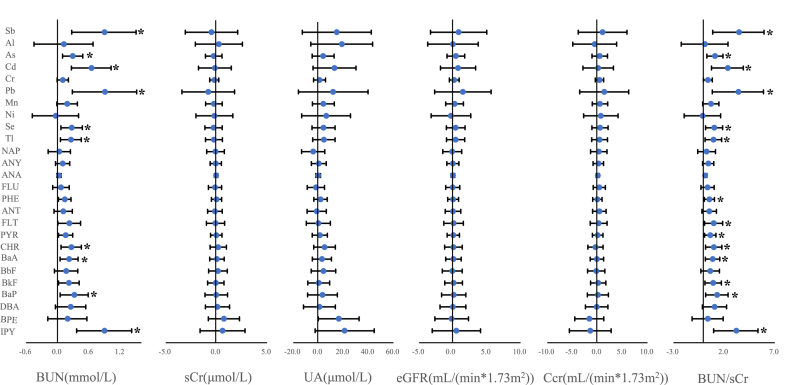
The cumulative changes (mean and 95% confidence intervals) in renal function indicators associated with an interquartile range increment of 3-day moving average of PM_2.5_-bound components. Abbreviations same as in Table 2 and Fig. 1. *Estimated were statistically significant (*p*-value < 0.05).

Fig. 2 illustrated the estimated changes in renal function indicators altered by ave 0–3 concentrations of trace metals and PAHs in PM_2.5_. Among the 10 metal (metalloid) constituents, short-term exposure to Sb, As, Cd, Pb, Se and Tl were related to increased BUN and BUN/sCr. For example, a IQR increment in Cd (1.02 ng/m^3^) was associated with 0.65 mmol/L increase in BUN (95% CI: 0.26 to 1.02) and 2.36 increase in BUN/sCr (95% CI: 0.75 to 3.89). The effects of each IQR increment in Pb (47.72 ng/m^3^) on BUN and BUN/sCr were 0.90 mmol/L (95% CI: 0.28 to 1.51) and 3.42 (95% CI: 0.84 to 5.89), respectively. Besides, PAHs of Chrysene, Benzo (a) anthracene, Benzo (a) pyrene and IPY in PM_2.5_ were also positively associated with BUN and BUN/sCr. For example, an IQR increment in IPY (0.30 ng/m^3^) were associated with 0.90 mmol/L (95% CI: 0.36 to 1.41) and 3.21 (95% CI: 0.97 to 5.34) higher of BUN and BUN/sCr, respectively. The relationships between the other 4 renal function indicators and the 26 PM_2.5_ constituents were insignificant.

### 3.4 Mediation analysis

**Fig. 3:**
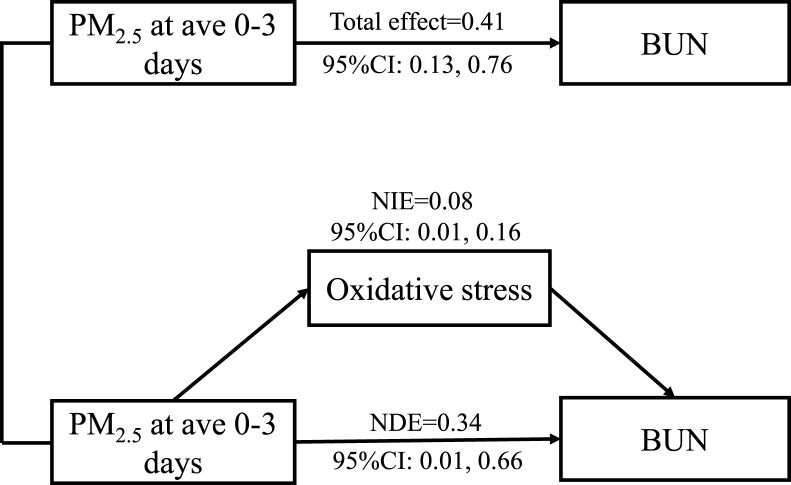
Mediation analysis of oxidative stress activation on blood urea nitrogen concentrations after PM_2.5_ exposure. Abbreviations: CI, confidence intervals; BUN, blood urea nitrogen; NIE, nature indirect effect; NDE, nature direct effect.

We explored the association between PM_2.5_ and potential mediators through LME models (Table 3). Each IQR increments in PM_2.5_ were associated with 1.64 U/mL (95% CI: 0.08 to 3.20) and 2.40 U/mL (95% CI: 0.37 to 4.42) increase in SOD at ave 0–2 and ave 0–3, respectively. The effects of PM_2.5_ exposure on IL-6, hsCRP, ACE and FIB were insignificant. Therefore, we further examined whether SOD could be a mediator of the associations between PM_2.5_ and renal function. It was estimated that SOD contributed to 18.24% of the associations between PM_2.5_ exposure and increased BUN at ave 0–3 (Fig. 3). Specifically, the NIE of PM_2.5_ (each 32.94 μg/m^3^ increment) on BUN was 0.08 mmol/L (95% CI: 0.01 to 0.16) at ave 0–3, while the NDE of PM_2.5_ was 0.34 mmol/L (95% CI: 0.01 to 0.66).

**Table 3:** Changes in potential mediators (mean and 95% confidence intervals) associated with an interquartile range increment of PM_2.5_ in different exposure windows.

	ave 0–1 days	ave 0–2 days	ave 0–3 days
SOD	1.51 (−0.22, 3.24)	1.64 (0.08, 3.20)*	2.40 (0.37, 4.42)*
IL-6	−0.05 (−0.33, 0.22)	−0.06 (−0.30, 0.19)	−0.09 (−0.37, 0.19)
hsCRP	−0.06 (−0.18, 0.07)	−0.05 (−0.16, 0.05)	−0.07 (−0.20, 0.06)
FIB	0.00 (−0.05, 0.04)	−0.01 (−0.05, 0.03)	−0.01 (−0.05, 0.03)
ACE	0.14 (−0.49, 0.75)	0.14 (−0.40, 0.68)	0.21 (−0.42, 0.84)

*Estimated were statistically significant (*p*-value < 0.05). Abbreviation: SOD, superoxide dismutase; FIB, fibrinogen; hsCRP, hypersensitive C-reactive protein; ACE, angiotensin converting enzyme; IL-6, interleukin-6.

### 3.5 Sensitivity analysis

A series of sensitivity analyses were conducted to check the robustness of our results. Firstly, we examined the time-varying confounding assumption using LME models, and found that there was insignificant association between *BUN*_*ij*_ and *SOD*_*ij+1*_ (Table S1). Additionally, we adjusted the other gaseous air pollutants into the two-pollutant LME models (Fig. S3), and the positive associations of PM_2.5_ with BUN and BUN/sCr were significant. Moreover, we adjusted the PM_2.5_ in the “constituent-PM_2.5_ joint models” (Fig. S4A) and controlled the residual constituents of the total mass concentrations in the “constituent-PM_2.5_ residual models” (Fig. S4B). We found relatively robust association between As, Cd, Pb, Se, Tl and IPY on BUN levels in both of two models.

## 4 Discussion

The research evaluated the adverse effect of PM_2.5_ and its trace constituents on renal function parameters over a period of 3 days among 35 healthy young adults. After adjusting for several potential covariates, PM_2.5_ mass concentrations and its several metals (metalloids) and PAHs showed strong associations with increased BUN and BUN/sCr. Additionally, we found elevated SOD levels due to PM_2.5_ exposure may mediate the association between PM_2.5_ exposure and renal functions. Our findings indicate that short-term exposure to PM_2.5_ may increase the risks of renal dysfunction via systemic oxidative stress, in which the constituents of Pb, Cd, As, Se, Tl and IPY may play the leading roles.

Numerous studies provided epidemiological evidence that PM_2.5_ may serve as a risk factor of renal dysfunction (Mehta et al., 2016; Tavera Busso et al., 2018; Liu et al., 2020; Rahmani Sani et al., 2020; Zhao et al., 2020; Li et al., 2021). Most studies reported the significant associations between PM_2.5_ and the kidney indicators of sCr and eGFR among populations. A recent panel study on 135 children aged 4–13 years reported that a 10 μg/m^3^ increment of PM_2.5_ was related to −1.83% changes in eGFR (Liu et al., 2020). The VA Normative Aging Study reported that per 2.1 μg/m^3^ increment in annual average of PM_2.5_ was related with 1.87 mL/min/1.73 m^2^ declination of eGFR among 669 older adults with an average age of 73.5 (Mehta et al., 2016). Another research reported that exposure to PM_2.5_ has a negative impact on renal function with 0.03 mg/dL increase in sCr and 1.09 mL/min/1.73 m^2^ reduction in eGFR among 150 pregnant women (Rahmani Sani et al., 2020). However, the associations of sCr and eGFR with PM_2.5_ were insignificant in this current research. This might be explained that children, the elderly and pregnant women were more vulnerable to the acute effects of PM_2.5_ on kidney than the healthy young adults in this study (Peled, 2011; Mukherjee and Agrawal, 2018).

The BUN, an end product of protein metabolism, was synthesized from amino acid metabolites in the liver and excreted from kidney. A cross-sectional study on pregnant women reported that for per 3.9 μg/m^3^ increment of PM_2.5_ was associated with 0.05 mmol/L increase in BUN during the whole pregnancy (Zhao et al., 2020). Additionally, an experimental study on rodent models reported that sub-chronic exposure to PM_2.5_ was related to BUN elevation (Tavera Busso et al., 2018). Our results were consistent with the findings of the above two research. As well-recognized indicators to reflect renal function, the concentrations of BUN and sCr are determined by the balance of body generation and excretion by the kidneys (Kirtane et al., 2005). However, BUN is partly reabsorbed by proximal tubules with sodium and water under the influence of antidiuretic hormone (Conte et al., 1987), whereas sCr is not (Matsue et al., 2017). Our research indicated that exposure to PM_2.5_ may be related to the elevated BUN levels without directly affecting glomerular filtration, which in turn increases the burden of renal function. The results can be explained that the elevated BUN levels may be due to renal hypoperfusion or tubular dysfunction, which was independent on the changes of eGFR and sCr (Aronson et al., 2004). Moreover, previous research reported that oxidative stress and inflammatory responses might increase the catabolism of structural proteins and amino acids, which in turn caused increases of BUN generation (Macedo, 2011).

PM_2.5_ contains a mixture of metallic/metalloid elements, adsorbed organic compounds and trace amounts of biological components (Bell et al., 2007), which varies in different regions. The heterogeneous constituents may explain the inconsistent results on the health effects of PM_2.5_ from numerous research. Because previous studies have reported the nephrotoxicity of several metals, we focused on the metal components of PM_2.5_ to estimate their effects on kidney (Navas-Acien et al., 2009; Trzeciakowski et al., 2014; Tsai et al., 2021). Regarding the PM_2.5_-bound metals, a study among 76 old participants reported that exposure to copper, titanium, and Mn in PM_2.5_ within 3 days were related to reduced eGFR (Fang et al., 2020). Another panel study in 144 children demonstrated that Mg^+^, K^+^, Al^+^ and Li^+^ in size-fractionated particle number counts of 0.5 were linked to eGFR reduction (Liu et al., 2021). In this study, we found Sb, Cd, Pb, Tl and metalloids of As, Se in PM_2.5_ were significantly related to elevated levels of BUN. Our results could be supported by a published research reporting that co-exposure to As, Pb, Cd, and Hg measured in urine was associated with increased BUN (Sanders et al., 2019). Additionally, we found weak but robust associations between elevated BUN levels and several PM_2.5_-bound PAHs. Generally, Pb and Sb in PM_2.5_ are originated from vehicle emissions (Smichowski et al., 2007), and PAHs are mainly from the incomplete combustion of fossil fuels (Ravindra et al., 2008). Therefore, our research suggested that PM_2.5_ from traffic sources may have greater effects on renal function.

Currently, the underlying biological mechanisms of the association between PM_2.5_ and renal function were not well characterized. One mechanistic hypothesis suggested that inhaled PM_2.5_ could stimulate the systematic inflammatory response and those inflammatory cytokines may impair the kidney via blood circulation (Rückerl et al., 2014; Suárez-Álvarez et al., 2016). In addition, the ultrafine particles may traverse the alveolar space into bloodstream and causes fibrinolytic dysfunction and cellular responses to exacerbate the damage of remote organs (Bowe et al., 2017; Xie et al., 2021). In this study, we measured several biomarkers of inflammation, oxidative stress, and vasoconstriction to exam their bio-mediation effects between PM_2.5_ and renal function parameters. This study found that SOD might be a potential mediator of the association between 3-days exposure of PM_2.5_ and BUN. Our results were consistent with existing evidence. A panel study reported that elevated SOD concentrations as an adaptive response of the organism to the oxidative stress in response to PM_2.5_ exposure (Wu et al., 2016). The human nonmercaptalbumin, an oxidative stress biomarker, was also reported to be positively related with BUN and sCr (Masudo et al., 2017). Additionally, two published experimental studies on rat models reported that exposure to PM_2.5_ led to early kidney damage as a consequence of oxidative stress-antioxidant imbalance (Aztatzi-Aguilar et al., 2016, 2021). Our study provides population-based epidemiological evidence that exposure to PM_2.5_ may affect the renal function via systemic oxidative stress. Nevertheless, the results of the mediation analysis should be interpreted cautiously and further studies are needed to verify the causal relationship.

This study has several strengths. Firstly, the research used a quasi-experimental design of the air quality controls during the 7th CISM Military World Games, which was efficient for causal inference. In addition, since all the participants in this study were healthy young adults and had no history of chronic diseases, the potential confounding effects of medications and diseases could be excluded. Thirdly, we employed the mediation analysis to explore the potential bio-mechanisms between PM_2.5_ and renal function, which provided important epidemiological evidence for PM_2.5_ induced renal dysfunction.

There were also several limitations in this study. Firstly, participants in this study were only including healthy young adults and the sample size was small, therefore the extrapolation of the research findings may be limited. Further studies are needed to include larger sample size with general population from multiple cities. Secondly, measurement bias is inevitable as the measurements of PM_2.5_ constituents were based on measurements from the nearest ambient monitoring station. However, we do not expect the bias to be substantial as all participants lived and worked within Wuhan University School of Medicine (less than 0.2 km). Previous research suggested that the ambient measurement of PM_2.5_ constituents at fixed monitoring sites could adequately be used to predict the individual-level exposures (Lei et al., 2020). Thirdly, although the renal function parameters in our research were conventional biomarkers from blood which with commonly used in clinical diagnosis, more indicators of early impairment of renal function such as serum Cystatin C, Kidney injury molecule-1 and Neutrophil gelatinase-associated lipid carrier protein (van Veldhuisen et al., 2016) could be considered in the future research.

## 5 Conclusion

The current panel study provided the evidence that short-term exposure to PM_2.5_ may affect renal function among healthy young adults. Several metal (metalloid) and PAHs components of PM_2.5_, such as Pb, Cd, As, Se, Tl and IPY, might contribute to the observed association. Additionally, our findings suggested that oxidative stress may be a plausible pathway which mediate the association between PM_2.5_ and BUN. The adverse effect of PM_2.5_, especially traffic-related particulate matters on renal function should be given more attention. Further studies are needed to verify our findings and elucidate the underlying mechanisms.

## Author contribution

Shouxin Peng: Data curation, Methodology, Formal analysis, Writing – original draft, Visualization. Tianjun Lu: Validation, Writing – review & editing. Yisi Liu: Validation, Writing – review & editing. Zhaoyuan Li: Methodology, Investigation, Software. Feifei Liu: Investigation, Data curation, Software. Jinhui Sun: Data curation, Software. Meijin Chen: Investigation. Huaiji Wang: Conceptualization, Investigation, Methodology. Hao Xiang: Conceptualization, Methodology, Writing – review & editing, Funding acquisition.

## Declaration of competing interest

The authors declare that they have no known competing financial interests or personal relationships that could have appeared to influence the work reported in this paper.

## References

[bib1] Al-Aly Z., Bowe B. (2020). Air pollution and kidney disease. Clin. J. Am. Soc. Nephrol..

[bib2] Aronson D., Mittleman M.A., Burger A.J. (2004). Elevated blood urea nitrogen level as a predictor of mortality in patients admitted for decompensated heart failure. Am. J. Med..

[bib3] Aztatzi-Aguilar O.G., Pardo-Osorio G.A., Uribe-Ramírez M., Narváez-Morales J., De Vizcaya-Ruiz A., Barbier O.C. (2021). Acute kidney damage by PM(2.5) exposure in a rat model. Environ. Toxicol. Pharmacol..

[bib4] Aztatzi-Aguilar O.G., Uribe-Ramirez M., Narvaez-Morales J., De Vizcaya-Ruiz A., Barbier O. (2016). Early kidney damage induced by subchronic exposure to PM2.5 in rats. Part. Fibre Toxicol..

[bib5] Bell M.L., Dominici F., Ebisu K., Zeger S.L., Samet J.M. (2007). Spatial and temporal variation in PM(2.5) chemical composition in the United States for health effects studies. Environ. Health Perspect..

[bib6] Bind M.A., Vanderweele T.J., Coull B.A., Schwartz J.D. (2016). Causal mediation analysis for longitudinal data with exogenous exposure. Biostatistics.

[bib7] Bowe B., Artimovich E., Xie Y., Yan Y., Cai M., Al-Aly Z. (2020). The global and national burden of chronic kidney disease attributable to ambient fine particulate matter air pollution: a modelling study. BMJ Glob Health.

[bib8] Bowe B., Xie Y., Li T., Yan Y., Xian H., Al-Aly Z. (2017). Associations of ambient coarse particulate matter, nitrogen dioxide, and carbon monoxide with the risk of kidney disease: a cohort study. The Lancet Planetary Health.

[bib9] Chenxu G., Minxuan X., Yuting Q., Tingting G., Jinxiao L., Mingxing W., Sujun W., Yongjie M., Deshuai L., Qiang L., Linfeng H., Jun T. (2018). iRhom2 loss alleviates renal injury in long-term PM2.5-exposed mice by suppression of inflammation and oxidative stress. Redox Biol..

[bib10] Cockcroft D.W., Gault M.H. (1976). Prediction of creatinine clearance from serum creatinine. Nephron.

[bib11] Conte G., Dal Canton A., Terribile M., Cianciaruso B., Di Minno G., Pannain M., Russo D., Andreucci V.E. (1987). Renal handling of urea in subjects with persistent azotemia and normal renal function. Kidney Int..

[bib12] Correia-Costa L., Sousa T., Morato M., Cosme D., Afonso J., Areias J.C., Schaefer F., Guerra A., Afonso A.C., Azevedo A., Albino-Teixeira A. (2016). Oxidative stress and nitric oxide are increased in obese children and correlate with cardiometabolic risk and renal function. Br. J. Nutr..

[bib13] Couser W.G., Remuzzi G., Mendis S., Tonelli M. (2011). The contribution of chronic kidney disease to the global burden of major noncommunicable diseases. Kidney Int..

[bib14] Eknoyan G., Lameire N., Barsoum R., Eckardt K.U., Levin A., Levin N., Locatelli F., MacLeod A., Vanholder R., Walker R., Wang H. (2004). The burden of kidney disease: improving global outcomes. Kidney Int..

[bib15] Ene-Iordache B., Perico N., Bikbov B., Carminati S., Remuzzi A., Perna A., Islam N., Bravo R.F., Aleckovic-Halilovic M., Zou H., Zhang L., Gouda Z., Tchokhonelidze I., Abraham G., Mahdavi-Mazdeh M., Gallieni M., Codreanu I., Togtokh A., Sharma S.K., Koirala P., Uprety S., Ulasi I., Remuzzi G. (2016). Chronic kidney disease and cardiovascular risk in six regions of the world (ISN-KDDC): a cross-sectional study. Lancet Global Health.

[bib16] Fang J., Tang S., Zhou J., Zhou J., Cui L., Kong F., Gao Y., Shen Y., Deng F., Zhang Y., Liu Y., Dong H., Dong X., Dong L., Peng X., Cao M., Wang Y., Ding C., Du Y., Wang Q., Wang C., Zhang Y., Wang Y., Li T., Shi X. (2020). Associations between personal PM(2.5) elemental constituents and decline of kidney function in older individuals: the China BAPE study. Environ. Sci. Technol..

[bib17] Kirtane A.J., Leder D.M., Waikar S.S., Chertow G.M., Ray K.K., Pinto D.S., Karmpaliotis D., Burger A.J., Murphy S.A., Cannon C.P., Braunwald E., Gibson C.M., Group T.S. (2005). Serum blood urea nitrogen as an independent marker of subsequent mortality among patients with acute coronary syndromes and normal to mildly reduced glomerular filtration rates. J. Am. Coll. Cardiol..

[bib18] Ko G.J., Rhee C.M., Kalantar-Zadeh K., Joshi S. (2020). The effects of high-protein diets on kidney health and longevity. J. Am. Soc. Nephrol..

[bib19] Kuźma Ł., Małyszko J., Bachórzewska-Gajewska H., Kralisz P., Dobrzycki S. (2021). Exposure to air pollution and renal function. Sci. Rep..

[bib20] Lei X., Chen R., Wang C., Shi J., Zhao Z., Li W., Bachwenkizi J., Ge W., Sun L., Li S., Cai J., Kan H. (2020). Necessity of personal sampling for exposure assessment on specific constituents of PM(2.5): results of a panel study in Shanghai, China. Environ. Int..

[bib21] Levey A.S., Stevens L.A., Schmid C.H., Zhang Y.L., Castro A.F., Feldman H.I., Kusek J.W., Eggers P., Van Lente F., Greene T., Coresh J. (2009). A new equation to estimate glomerular filtration rate. Ann. Intern. Med..

[bib22] Li Q., Wang Y.Y., Guo Y., Zhou H., Wang Q.M., Shen H.P., Zhang Y.P., Yan D.H., Li S., Chen G., Lin L., He Y., Yang Y., Peng Z.Q., Wang H.J., Ma X. (2021). Association between airborne particulate matter and renal function: an analysis of 2.5 million young adults. Environ. Int..

[bib23] Li Z., Liu Q., Xu Z., Guo X., Wu S. (2020). Association between short-term exposure to ambient particulate air pollution and biomarkers of oxidative stress: a meta-analysis. Environ. Res..

[bib24] Liu C., Cai J., Qiao L., Wang H., Xu W., Li H., Zhao Z., Chen R., Kan H. (2017). The acute effects of fine particulate matter constituents on blood inflammation and coagulation. Environ. Sci. Technol..

[bib25] Liu M., Guo W., Cai Y., Yang H., Li W., Yang L., Lai X., Fang Q., Ma L., Zhu R., Zhang X. (2020). Personal exposure to fine particulate matter and renal function in children: a panel study. Environ. Pollut..

[bib26] Liu M., Guo W., Yang H., Zhao L., Fang Q., Li M., Shu J., Jiang Y., Lai X., Yang L., Zhang X. (2021). Short-term effects of size-fractionated particulate matters and their constituents on renal function in children: a panel study. Ecotoxicol. Environ. Saf..

[bib27] Lue S.H., Wellenius G.A., Wilker E.H., Mostofsky E., Mittleman M.A. (2013). Residential proximity to major roadways and renal function. J. Epidemiol. Community Health.

[bib28] Macedo E. (2011). Blood urea nitrogen beyond estimation of renal function. Crit. Care Med..

[bib29] Mao X., Hu X., Wang Y., Xia W., Zhao S., Wan Y. (2020). Temporal trend of arsenic in outdoor air PM2.5 in Wuhan, China, in 2015-2017 and the personal inhalation of PM-bound arsenic: implications for human exposure. Environ. Sci. Pollut. Res. Int..

[bib30] Masudo R., Yasukawa K., Nojiri T., Yoshikawa N., Shimosaka H., Sone S., Oike Y., Ugawa A., Yamazaki T., Shimokado K., Yatomi Y., Ikeda H. (2017). Evaluation of human nonmercaptalbumin as a marker for oxidative stress and its association with various parameters in blood. J. Clin. Biochem. Nutr..

[bib31] Matsue Y., van der Meer P., Damman K., Metra M., O'Connor C.M., Ponikowski P., Teerlink J.R., Cotter G., Davison B., Cleland J.G., Givertz M.M., Bloomfield D.M., Dittrich H.C., Gansevoort R.T., Bakker S.J., van der Harst P., Hillege H.L., van Veldhuisen D.J., Voors A.A. (2017). Blood urea nitrogen-to-creatinine ratio in the general population and in patients with acute heart failure. Heart.

[bib32] Mehta A.J., Zanobetti A., Bind M.A., Kloog I., Koutrakis P., Sparrow D., Vokonas P.S., Schwartz J.D. (2016). Long-term exposure to ambient fine particulate matter and renal function in older men: the veterans administration normative aging study. Environ. Health Perspect..

[bib33] Mukherjee A., Agrawal M. (2018). A global perspective of fine particulate matter pollution and its health effects. Rev. Environ. Contam. Toxicol..

[bib34] Navas-Acien A., Tellez-Plaza M., Guallar E., Muntner P., Silbergeld E., Jaar B., Weaver V. (2009). Blood cadmium and lead and chronic kidney disease in US adults: a joint analysis. Am. J. Epidemiol..

[bib35] Peled R. (2011). Air pollution exposure: who is at high risk?. Atmos. Environ..

[bib36] Peng S., Sun J., Liu F., Li Z., Wu C., Xiang H. (2022). The effect of short-term fine particulate matter exposure on glucose homeostasis: a panel study in healthy adults. Atmos. Environ..

[bib37] Rahmani Sani A., Abroudi M., Heydari H., Adli A., Miri M., Mehrabadi S., Pajohanfar N.S., Raoufinia R., Bazghandi M.S., Ghalenovi M., Rad A., Miri M., Dadvand P. (2020). Maternal exposure to ambient particulate matter and green spaces and fetal renal function. Environ. Res..

[bib38] Ravindra K., Sokhi R., Vangrieken R. (2008). Atmospheric polycyclic aromatic hydrocarbons: source attribution, emission factors and regulation. Atmos. Environ..

[bib39] Rückerl R., Hampel R., Breitner S., Cyrys J., Kraus U., Carter J., Dailey L., Devlin R.B., Diaz-Sanchez D., Koenig W., Phipps R., Silbajoris R., Soentgen J., Soukup J., Peters A., Schneider A. (2014). Associations between ambient air pollution and blood markers of inflammation and coagulation/fibrinolysis in susceptible populations. Environ. Int..

[bib40] Sanders A.P., Mazzella M.J., Malin A.J., Hair G.M., Busgang S.A., Saland J.M., Curtin P. (2019). Combined exposure to lead, cadmium, mercury, and arsenic and kidney health in adolescents age 12-19 in NHANES 2009-2014. Environ. Int..

[bib41] Smichowski P., Gómez D., Frazzoli C., Caroli S. (2007). Traffic‐related elements in airborne particulate matter. Appl. Spectrosc. Rev..

[bib42] Stenvinkel P., Shiels P.G., Painer J., Miranda J.J., Natterson-Horowitz B., Johnson R.J. (2020). A planetary health perspective for kidney disease. Kidney Int..

[bib43] Suárez-Álvarez B., Liapis H., Anders H.J. (2016). Links between coagulation, inflammation, regeneration, and fibrosis in kidney pathology. Lab. Invest..

[bib44] Tang H., Cheng Z., Li N., Mao S., Ma R., He H., Niu Z., Chen X., Xiang H. (2020). The short- and long-term associations of particulate matter with inflammation and blood coagulation markers: a meta-analysis. Environ. Pollut..

[bib45] Tavera Busso I., Mateos A.C., Juncos L.I., Canals N., Carreras H.A. (2018). Kidney damage induced by sub-chronic fine particulate matter exposure. Environ. Int..

[bib46] Trzeciakowski J.P., Gardiner L., Parrish A.R. (2014). Effects of environmental levels of cadmium, lead and mercury on human renal function evaluated by structural equation modeling. Toxicol. Lett..

[bib47] Tsai H.J., Hung C.H., Wang C.W., Tu H.P., Li C.H., Tsai C.C., Lin W.Y., Chen S.C., Kuo C.H. (2021). Associations among heavy metals and proteinuria and chronic kidney disease. Diagnostics.

[bib48] Upadhyay A., Larson M.G., Guo C.Y., Vasan R.S., Lipinska I., O'Donnell C.J., Kathiresan S., Meigs J.B., Keaney J.F., Rong J., Benjamin E.J., Fox C.S. (2011). Inflammation, kidney function and albuminuria in the Framingham Offspring cohort. Nephrol. Dial. Transplant..

[bib49] Valeri L., Vanderweele T.J. (2013). Mediation analysis allowing for exposure-mediator interactions and causal interpretation: theoretical assumptions and implementation with SAS and SPSS macros. Psychol. Methods.

[bib50] van Veldhuisen D.J., Ruilope L.M., Maisel A.S., Damman K. (2016). Biomarkers of renal injury and function: diagnostic, prognostic and therapeutic implications in heart failure. Eur. Heart J..

[bib51] Wei Y., Han I.K., Shao M., Hu M., Zhang O.J., Tang X. (2009). PM2.5 constituents and oxidative DNA damage in humans. Environ. Sci. Technol..

[bib52] Winter M.A., Guhr K.N., Berg G.M. (2012). Impact of various body weights and serum creatinine concentrations on the bias and accuracy of the Cockcroft-Gault equation. Pharmacotherapy.

[bib53] Wu M.Y., Lo W.C., Chao C.T., Wu M.S., Chiang C.K. (2020). Association between air pollutants and development of chronic kidney disease: a systematic review and meta-analysis. Sci. Total Environ..

[bib54] Wu S., Deng F., Niu J., Huang Q., Liu Y., Guo X. (2011). Exposures to PM₂.₅ components and heart rate variability in taxi drivers around the Beijing 2008 Olympic Games. Sci. Total Environ..

[bib55] Wu S., Wang B., Yang D., Wei H., Li H., Pan L., Huang J., Wang X., Qin Y., Zheng C., Shima M., Deng F., Guo X. (2016). Ambient particulate air pollution and circulating antioxidant enzymes: a repeated-measure study in healthy adults in Beijing, China. Environ. Pollut..

[bib56] Xie W., You J., Zhi C., Li L. (2021). The toxicity of ambient fine particulate matter (PM2.5) to vascular endothelial cells. J. Appl. Toxicol..

[bib57] Xu X., Nie S., Ding H., Hou F.F. (2018). Environmental pollution and kidney diseases. Nat. Rev. Nephrol..

[bib58] Yilmaz M.I., Saglam M., Caglar K., Cakir E., Sonmez A., Ozgurtas T., Aydin A., Eyileten T., Ozcan O., Acikel C., Tasar M., Genctoy G., Erbil K., Vural A., Zoccali C. (2006). The determinants of endothelial dysfunction in CKD: oxidative stress and asymmetric dimethylarginine. Am. J. Kidney Dis..

[bib59] Zhang L., Wang F., Wang L., Wang W., Liu B., Liu J., Chen M., He Q., Liao Y., Yu X., Chen N., Zhang J.E., Hu Z., Liu F., Hong D., Ma L., Liu H., Zhou X., Chen J., Pan L., Chen W., Wang W., Li X., Wang H. (2012). Prevalence of chronic kidney disease in China: a cross-sectional survey. Lancet.

[bib60] Zhao Y., Cai J., Zhu X., van Donkelaar A., Martin R.V., Hua J., Kan H. (2020). Fine particulate matter exposure and renal function: a population-based study among pregnant women in China. Environ. Int..

