# Supplementary material for: Short-term exposure to fine particulate matter and its constituents may affect renal function via oxidative stress: A longitudinal panel study
Source: Chemosphere. 2022 Apr;293:133570. doi: 10.1016/j.chemosphere.2022.133570 (PMC8976286; doi:10.1016/j.chemosphere.2022.133570)
Supplement: Multimedia component 1 [file mmc1.pdf]

Supplemental Material

Short-term exposure to fine particulate matter and its constituents may affect renal function via oxidative stress: A longitudinal panel study

Table of contents

Table S1. Changes in superoxide dismutase (mean and 95% confidence intervals) with increase in [BUN blood urea nitrogen (mmol/L)] concentrations at previous visits (Y_{ij} → M_{ij+1}).

M_{ij+1}	Changes and 95% confidence	*p*-value
Superoxide dismutase	-0.55 (-1.66, 0.56)	0.37

Figure of contents

Figure S1. Directed acyclic graph for mediation analysis. $PM_{2.5}^{ij}$ represents $PM_{2.5}$ exposure for i^{th} subject prior to j^{th} visit; $M^{i,j}$ represents the potential bio-mediator levels for i^{th} subject prior to j^{th} visit; $Y^{i,j}$ represents the renal function indicators for i^{th} subject prior to j^{th} visit; $C_1^{i,j}$ represents exposure-outcome confounders; $C_2^{i,j}$ represents exposure-mediator confounders; $C_3^{i,j}$ represent mediator-outcome confounders. The dotted line indicates the time-varying confounding assumption.

Figure S2. Monthly averaged $PM_{2.5}$ concentrations from September to December in Wuhan, China, 2018 to 2020.

Figure S3. Changes in blood urea nitrogen and blood urea nitrogen-to-serum creatinine (mean and 95% confidence intervals) with an interquartile range increment of 3-day averaged $PM_{2.5}$ concentrations in the two-pollutant models.

Figure S4. Changes (mean and 95% confidence intervals) in blood urea nitrogen associated with an interquartile range increase in 3-day average concentrations of $PM_{2.5}$-bound constituents in the constituent-$PM_{2.5}$ joint models and the constituent-residual model. Abbreviations same as in Table 2. *Estimated were statistically significant (p-value < 0.05).

Figure S1

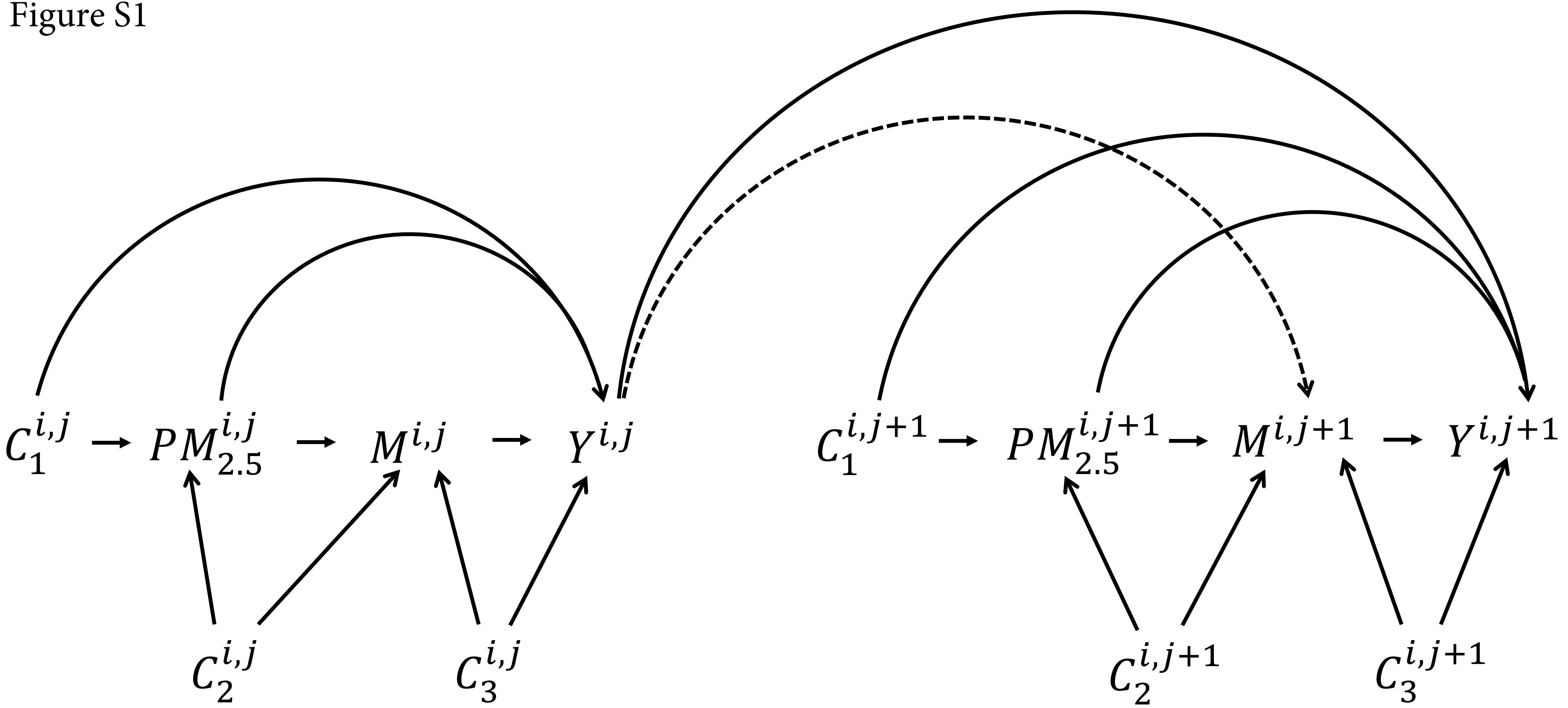

$C_1^{i,j}$
$PM_{2.5}^{i,j}$
$M^{i,j}$
$Y^{i,j}$
$C_2^{i,j}$
$C_3^{i,j}$
$C_1^{i,j+1}$
$PM_{2.5}^{i,j+1}$
$M^{i,j+1}$
$Y^{i,j+1}$
$C_2^{i,j+1}$
$C_3^{i,j+1}$

Figure S2

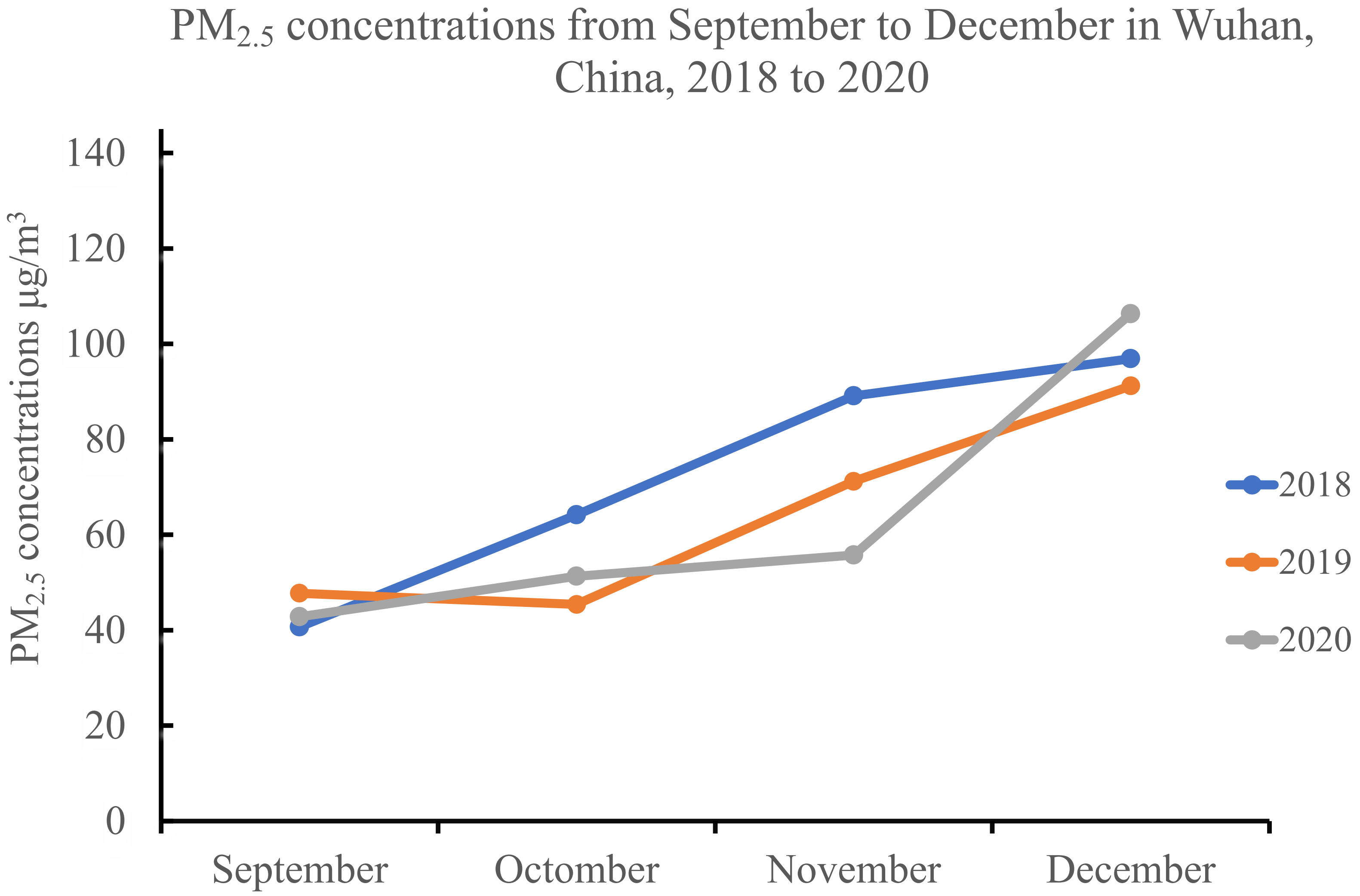

PM2.5 concentrations from September to December in Wuhan, China, 2018 to 2020
PM2.5 concentrations μg/m3
140
120
100
80
60
40
20
0
September
Octomber
November
December
2018
2019
2020

Figure S3

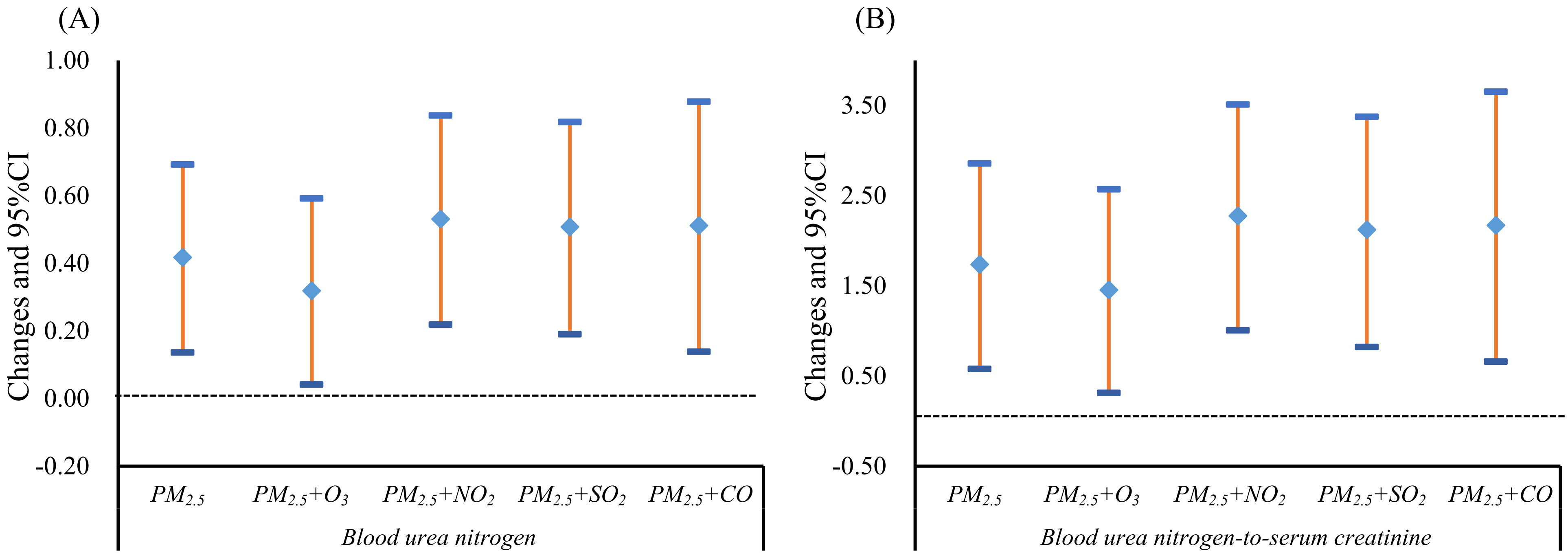

(A)
1.00
0.80
0.60
0.40
0.20
0.00
-0.20
Changes and 95%CI
PM2.5
PM2.5+O3
PM2.5+NO2
PM2.5+SO2
PM2.5+CO
Blood urea nitrogen
(B)
3.50
2.50
1.50
0.50
-0.50
Changes and 95%CI
PM2.5
PM2.5+O3
PM2.5+NO2
PM2.5+SO2
PM2.5+CO
Blood urea nitrogen-to-serum creatinine

Figure S4

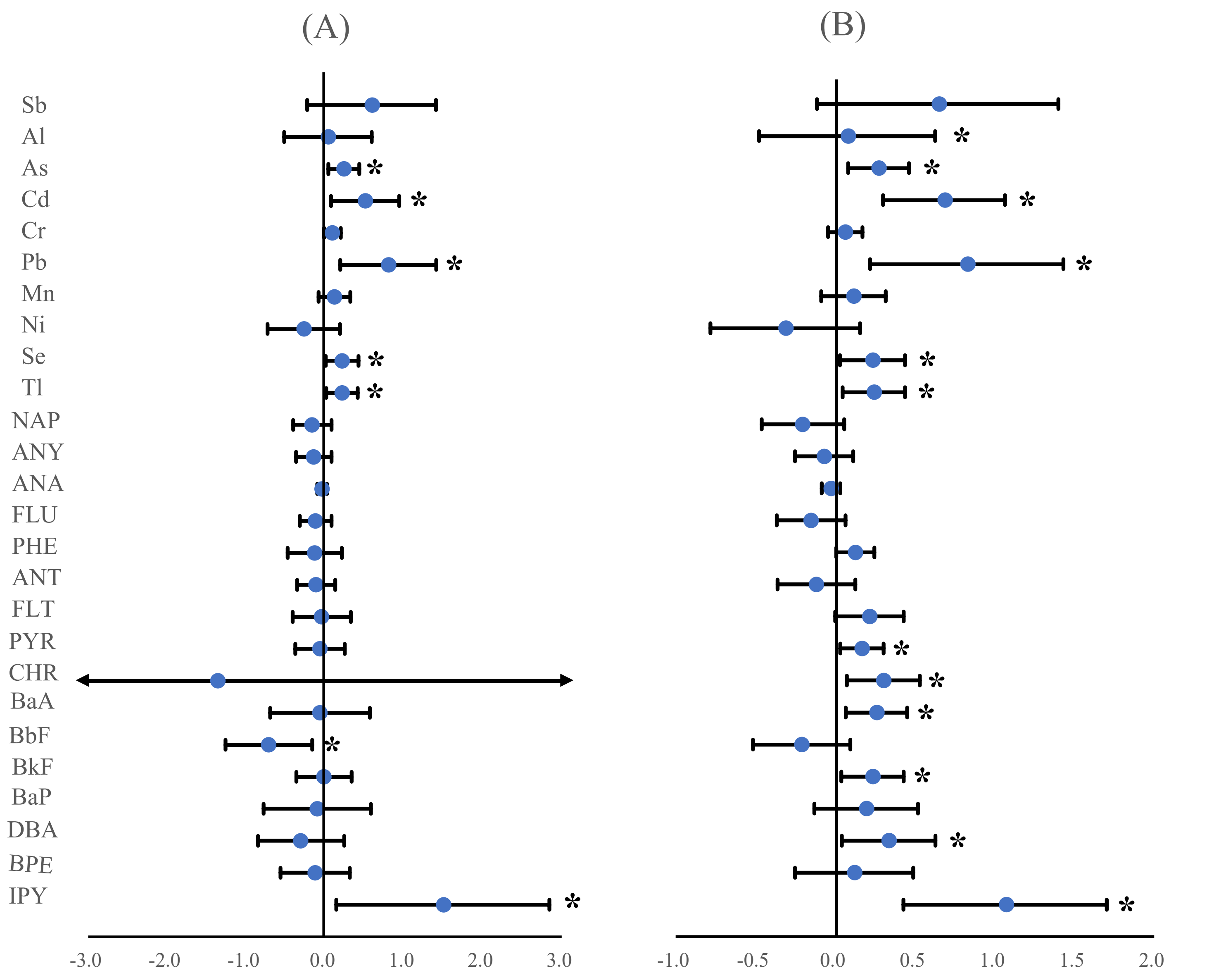

(A)
(B)
Sb
Al
As
Cd
Cr
Pb
Mn
Ni
Se
Tl
NAP
ANY
ANA
FLU
PHE
ANT
FLT
PYR
CHR
BaA
BbF
BkF
BaP
DBA
BPE
IPY
-3.0
-2.0
-1.0
0.0
1.0
2.0
3.0
-1.0
-0.5
0.0
0.5
1.0
1.5
2.0
